# Electron transfer studies of a conventional redox probe in human sweat and saliva bio-mimicking conditions

**DOI:** 10.1038/s41598-021-86866-z

**Published:** 2021-04-07

**Authors:** P. Krishnaveni, V. Ganesh

**Affiliations:** 1 Electrodics and Electrocatalysis (EEC) Division, CSIR – Central Electrochemical Research Institute (CSIR – CECRI), Karaikudi, 630003 Tamil Nadu India; 2 Academy of Scientific and Innovative Research (AcSIR), Ghaziabad, 201002 India

**Keywords:** Electrochemistry, Surface chemistry, Soft materials

## Abstract

Modern day hospital treatments aim at developing electrochemical biosensors for early diagnosis of diseases using unconventional human bio-fluids like sweat and saliva by monitoring the electron transfer reactions of target analytes. Such kinds of health care diagnostics primarily avoid the usage of human blood and urine samples. In this context, here we have investigated the electron transfer reaction of a well-known and commonly used redox probe namely, potassium ferro/ferri cyanide by employing artificially simulated bio-mimics of human sweat and saliva as unconventional electrolytes. Typically, electron transfer characteristics of the redox couple, [Fe(CN)_6_]^3−/4−^ are investigated using electrochemical techniques like cyclic voltammetry and electrochemical impedance spectroscopy. Many different kinetic parameters are determined and compared with the conventional system. In addition, such electron transfer reactions have also been studied using a lyotropic liquid crystalline phase comprising of Triton X-100 and water in which the aqueous phase is replaced with either human sweat or saliva bio-mimics. From these studies, we find out the electron transfer reaction of [Fe(CN)_6_]^3−/4−^ redox couple is completely diffusion controlled on both Au and Pt disc shaped electrodes in presence of sweat and saliva bio-mimic solutions. Moreover, the reaction is partially blocked by the presence of lyotropic liquid crystalline phase consisting of sweat and saliva bio-mimics indicating the predominant charge transfer controlled process for the redox probe. However, the rate constant values associated with the electron transfer reaction are drastically reduced in presence of liquid crystalline phase. These studies are essentially carried out to assess the effect of sweat and saliva on the electrochemistry of Fe^2+/3+^ redox couple.

## Introduction

Electron transfer reactions play a vital role not only in chemical reactions, industrial processes but also in everyday aspects of human life. Biosensor is typically a device comprising of sensing element, transducer and the connected electronics. In most of the electrochemical biosensors, electron transfer reaction is monitored using electrochemical techniques. These sensors are basically used to identify and estimate small molecule analytes like glucose, hydrogen peroxide, NADH, hemoglobin, urea etc., especially the species of biological importance. Generally human body fluids like blood, plasma, serum and urine are used to detect such kinds of bio-analytes. Depending upon the structure of target bio-species either in-built redox reactions or external redox probe is used to monitor the electron transfer reactions. Usually, blood tests have been carried out for the confirmation of various metabolites blends in human body for the diagnosis and regardless, blood analyzing is genuinely prominent. Recent advances in this area of research indicate the possibility of using human sweat and saliva as an alternate, non-invasive yet powerful medium to detect and analyze many important bio-analytes^1–4^. These analytical methods are especially needed for patients who require keeping up day-to-day monitoring of certain parameters, for example, diabetes, blood pressure etc. Though many assessing areas are available for non-intrusive and non-invasive detection, sweat and salivation are easily accessible^1–4^. Interestingly, many potential bio-markers such as inorganic particles^5^, ethanol^6–8^, amino acids^9^, lactate^1^, angiotensin^10^, immunoglobulin-A^11^, peptide^12^, protein^13^, and interleukin-1a^14^ etc. present in human body fluids. In order to utilize the measure of these species as clinical parameters, we must know the characteristics of each species in sweat and ideally it should correlate with that of blood as well as providing information about individual's well-being condition.

Similarly, salivation is a staggering exocrine release found in vertebrates and creatures of land and water that contains ~ 2290 proteins. Salivation is a complex bio-fluid contains various constituents saturating from blood by means of transcellular or paracellular ways. Consequently, saliva-chemistry offers an amazing non-obtrusive option to blood examination for checking enthusiastic, hormonal, nourishing and metabolic condition of the human body^15–20^. Salivary sensors in principle, have concentrated on the non-invasive potentiometric monitoring of electrolytes, for example, fluoride^21^, pH^22^, and sodium^23^. There are a numerous distributions that propose utilizing human sweat and salivation rather than blood, for assessing metabolites present in sweat and salivation towards the development of electrochemical biosensors^17–20^. Many literature reports that deal with utilizing change in viscosity^24^, pH^25^, measurement of stress biomarkers like Cortisol^26^ and concentration of chloride ion^27^ in human body fluids are available. These studies clearly demonstrate the possibility of using sweat and saliva for health care diagnostics.

Electrochemical reactions are pivotal to many devices including electrolyzer, fuel cells, batteries, biosensors and so on. Electrochemical techniques usually provide insight information on the electron transfer reactions of molecules, atoms, ions occur at electrode–electrolyte interfaces. Further such information will be useful to assess the potential range of reactions, stability of intermediates and the significant thermodynamic data. In case of biological system, generally enzymes react with the target analyte species producing hydrogen peroxide as a by-product in most of the cases, which is measured using electrochemical methods^28^. A well-known and most popular redox couple namely, [Fe(CN)_6_]^3−/4−^ is widely used for the determination of various biological systems using both the electrochemical and spectroscopic techniques^29^. Kinetic parameters like diffusion coefficient values, electron transfer rate constants and charge transfer resistance are found to differ with the nature of electrolyte prepared using the conventional aqueous medium^30,31^. Interestingly the nature and purity of electrode surface is assessed with the aid of [Fe(CN)_6_]^3−/4−^ redox couple and the effect of composition of the electrode materials and surface pre-treatments like potential cycling on the kinetic parameters is also investigated^32,33^. Similarly the electron transfer kinetics of four different redox couples namely, [Fe(CN)_6_]^3−/4−^, [Ru(NH_3_)_6_]^2+/3+^, [IrCl_6_]^2+/3+^ and methyl viologen, (MV^0/2+^) are studied using polycrystalline boron-doped diamond thin-film electrode and the presence of oxygen moieties on the surface of the electrode is found to alter the characteristics of the redox couple^34^. Such kinds of redox probes are also utilized to investigate the electron transfer behaviour of chemically modified electrodes, especially for self-assembled monolayer (SAM) modified electrodes^35,36^. These studies found out that such electron transfer reactions mainly depend upon the nature of electrode surface, chemical composition of the deposited materials and the electrolyte medium etc. Therefore, the study of electron transfer reactions is nearly inevitable and herein we report such study of a conventional redox probe in an unconventional electrolytic medium.

**Scheme 1:**
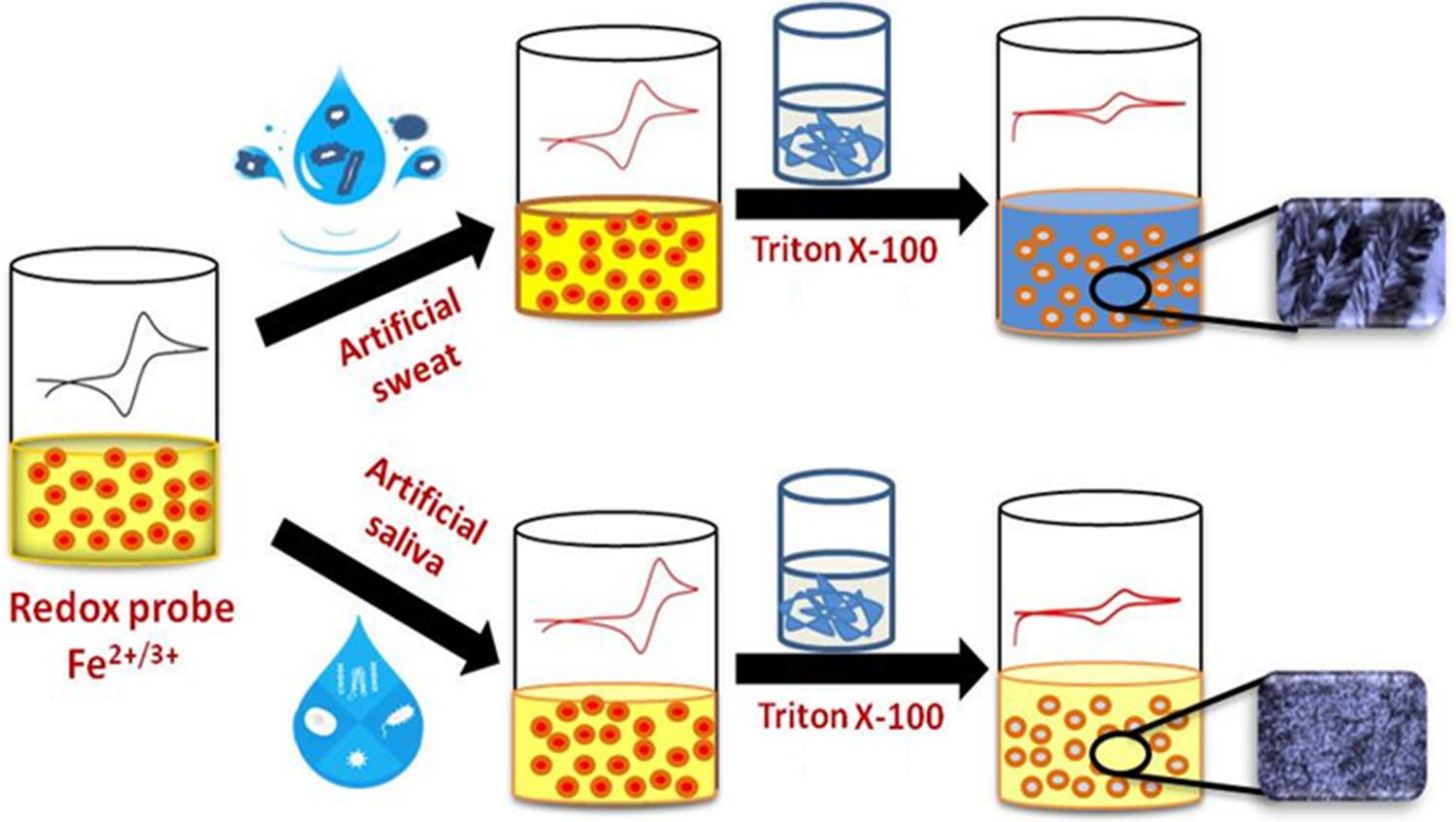
Pictorial representation of the investigation of Fe^2+/3+^ redox couple in unconventional electrolytes formed using artificial sweat and saliva compositions.

Particularly, in this work, we report the investigation of electron transfer reaction associated with a well-known redox probe, namely potassium ferro/ferri cyanide using artificially simulated human sweat and saliva solutions being used as the unconventional electrolytes. Primary objective of this work is to assess the effect of sweat and saliva compositions on the electrochemistry of Fe^2+/3+^ redox couple and to understand the factors that influence electron transfer reaction between the electrolytes (sweat and saliva) and the electrode (Au and Pt) surfaces (Scheme 1). In addition to this, similar electron transfer reactions are also studied using Triton X-100/water system that exhibits lyotropic liquid crystalline phase in which the aqueous component is replaced by either sweat or saliva bio-mimic solution (Scheme 1). The formation of hexagonal structure of the liquid crystalline phase consisting of the redox probe along with sweat and saliva compositions is confirmed by polarizing optical microscopy (POM) studies. Electrochemical techniques such as cyclic voltammetry (CV) and electrochemical impedance spectroscopy (EIS) are used to investigate such systems. Different characteristic parameters including diffusion coefficient, electron transfer rate constant and charge transfer resistance values are determined and compared with the values obtained for the conventional systems. Hence this particular work would provide better understanding of electron transfer reactions occurring in sweat and saliva bio-mimics that could lead to the development of viable electrochemical biosensors for the detection of potential biomarkers present in these bio-fluids. This will also provide a platform to correlate analysis of several diseases using such bio-fluids.

## Results and discussion

### Polarizing optical microscopic studies

**Figure 1:**
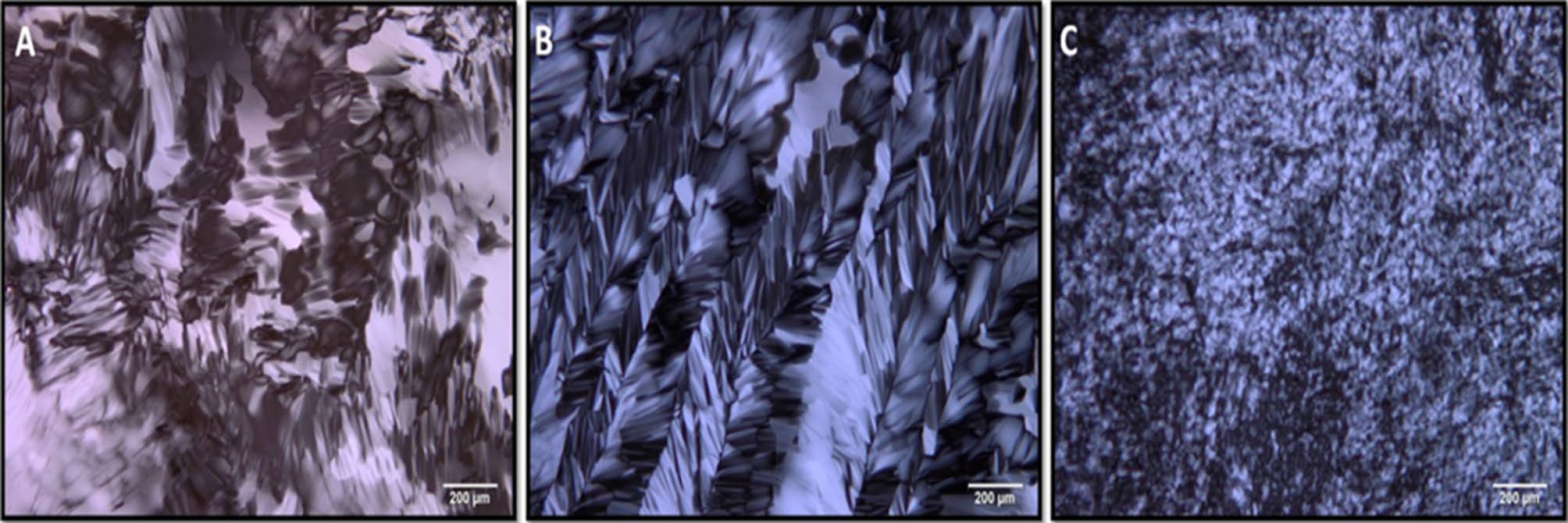
Polarizing optical microscopic images recorded for (**A**) Triton X-100 and aqueous solution consisting of 1 mM [Fe(CN)_6_]^3−/4−^, (**B**) Triton X-100 and [Fe(CN)_6_]^3−/4−^ along with artificial sweat solution and (**C**) Triton X-100 and [Fe(CN)_6_]^3−/4−^ along with saliva solution at a temperature of 25 °C respectively. These images clearly show the formation of hexagonal liquid crystalline phase for all these compositions.

Polarized optical microscopy (POM) is generally used to analyze the characteristics associated with phase transitions. Specifically, in this work POM is used to confirm the formation of liquid crystalline phase consisting of water (52 wt%) and Triton X-100 (48 wt%) compositions where the redox probe is dissolved in the aqueous medium. In case of artificially simulated bio-mimics, the aqueous phase in the above composition is replaced with either sweat or saliva constituents. POM analysis is a simple method to identify the formation of mesophases. In a typical experiment, appearance of textures is noted by sandwiching the sample between the glass slides and cover slips. If needed, temperature could also be varied using a temperature controller. In this work, textural changes were analyzed by varying the temperature and the corresponding images were recorded by cooling the samples. Figure 1 shows POM images of the textures obtained for various compositions such as hexagonal liquid crystalline phase with the redox probe (A), and a similar composition with artificially simulated sweat (B) and saliva (C) bio-mimics respectively. These images show the formation of typical broken focal conic like structure^37^ and striations^38^ corresponding to the hexagonal liquid crystalline phase. The ordered arrangement and the appearance of beautiful textures clearly indicate the formation of hexagonal liquid crystalline phase for these compositions. Initially in case of water and Triton X-100 mixture consisting of the redox probe, the formation of broken focal conic texture is clearly seen (Fig. 1A). Upon introducing sweat composition in the above liquid crystalline phase (Fig. 1B), the texture is changed to beautiful striations along with broken focal conic texture clearly indicating the prepared composition retains the liquid crystalline phase. On the other hand, addition of saliva constituents to the above mixture results in a peculiar texture where the small pieces of broken focal conic texture is observed (Fig. 1C). Basically the characteristic changes in birefringence at the phase transition are reflected upon as the textural changes pronounced through structural alternation of molecules in the liquid crystalline system. These studies clearly show the formation of hexagonal liquid crystalline phase displaying the polarizability changes of constituent molecules and retaining the same phase along with the liquid crystalline order even after the introduction of sweat and saliva compositions.

### Electrochemical studies using artificially simulated sweat and saliva bio-mimics

In this work, the fundamental electron transfer characteristics of a well-known redox probe, namely potassium ferro/ferri cyanide are investigated using unconventional electrolyte comprising of artificially simulated human sweat and saliva systems. Electrochemical techniques such as CV and EIS are used for the investigation of electron transfer reaction and for the subsequent determination of kinetic parameters under different conditions.

### Cyclic voltammetry

**Figure 2:**
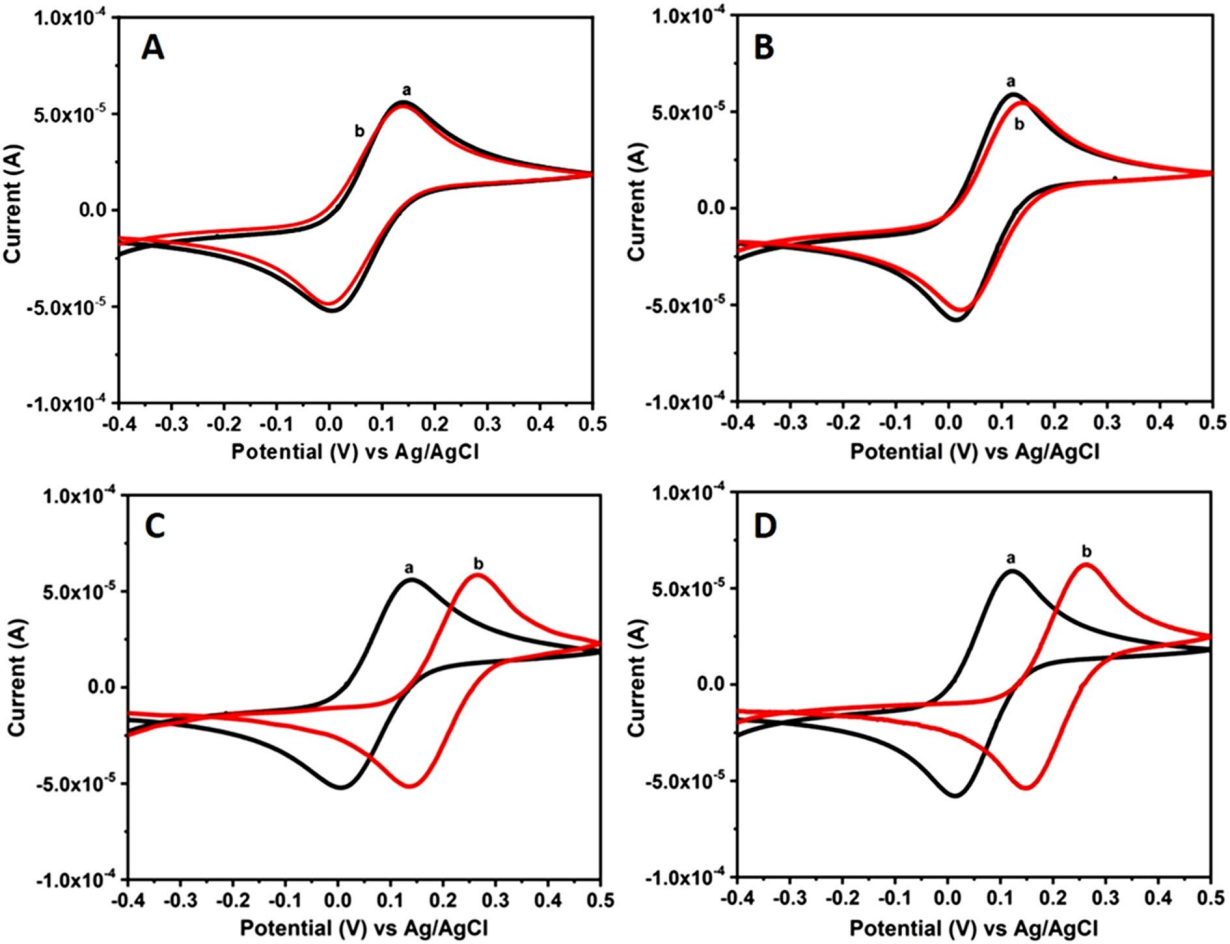
CV curves of Au and Pt electrodes recorded using sweat and saliva bio-mimic solutions at a fixed scan rate of 50 mV/s. (**A**) Au and (**B**) Pt electrodes in artificially simulated sweat solution + Fe^2+/3+^ redox couple. Similarly (**C**) Au and (**D**) Pt electrodes in artificial saliva solution + Fe^2+/3+^ redox couple. These CVs are recorded for 1 mM [Fe(CN)_6_]^3−/4−^ redox couple containing 0.1 M NaCl in absence (a) and in presence (b) of either sweat or saliva compositions respectively.

Cyclic voltammetric experiments are performed using unconventional electrolytes consisting of the bio-mimicking sweat and saliva compositions along with 1 mM [Fe(CN)_6_]^3−/4−^ as a redox couple and 0.1 M NaCl as a supporting electrolyte. These studies are carried out using Au and Pt as the working electrodes. The corresponding voltammograms were recorded by scanning within the potential ranging from − 0.4 to + 0.5 V (vs. Ag/AgCl) at a fixed scan rate of 50 mV/s and the resultant CVs are shown in Fig. 2. These CV curves were recorded using the electrolyte consisting of 1 mM [Fe(CN)_6_]^3−/4−^ in absence (‘a’ curves in Fig. 2) and in presence of either sweat or saliva compositions (‘b’ curves in Fig. 2). It can be noted from these CVs, the clear formation of redox peaks associated with Fe^2+/3+^ redox couple for both the electrodes indicating a reversible faradaic reaction occurring in case of both sweat and saliva compositions. For comparison similar CV curves were recorded with artificially simulated sweat and saliva compositions in the absence of redox probe (Electronic supporting information (ESI); Fig. S1). These CV curves show no distinguishable peak formation suggesting both the sweat and saliva compositions are electrochemically silent within the potential window of study. These results also indicate the absence of any faradaic redox reactions arising from such simulated compositions.

From these CV studies many different parameters including peak potential, peak current values for oxidation and reduction processes of the redox couple are determined. The extracted peak potential values (both for oxidation and reduction processes namely E_ox_ & E_red_), half-wave potential values (E_1/2_) and the peak current values (both for oxidation and reduction processes namely I_p_^ox^ & I_p_^red^) at a fixed scan rate of 50 mV/s along with pH of various systems investigated in this work are given in Table 1.

**Table 1:** pH, peak potential values (Oxidation—E_ox_; Reduction—E_red_), peak current values (Oxidation—I_p_^ox^; Reduction—I_p_^red^) and half-wave potential values (E_1/2_) at a scan rate of 50 mV/s for Au and Pt electrodes obtained from the CV curves recorded using [Fe(CN)_6_]^3−/4−^ redox couple for various sweat and saliva bio-mimic solutions studied in this work.

Electrolyte	pH	Peak potentials (mV)				Peak currents (× 10^–5^ A)				E_1/2_ (mV)	
		Au		Pt		Au		Pt		Au	Pt
		E_ox_	E_red_	E_ox_	E_red_	I_p_^ox^	I_p_^red^	I_p_^ox^	I_p_^red^		
Fe^2+/3+^	7.3	141	05	119	12	5.5	− 5.2	5.8	− 5.7	136	107
Sweat + Fe^2+/3+^	6.6	135	02	139	22	5.8	− 5.2	5.4	− 5.2	133	117
Sweat + Fe^2+/3+^ + Triton X-100	5.9	144	28	126	25	1.3	− 1.2	1.4	− 1.3	116	101
Saliva + Fe^2+/3+^	6.2	265	134	261	147	5.8	− 5.1	6.2	− 5.3	131	114
Saliva + Fe^2+/3+^ + Triton X-100	5.5	191	33	164	10	1.4	− 1.2	1.0	− 1.0	158	154
Real sweat sample analysis	6.5	133	03	137	25	5.7	− 5.1	5.3	− 5.1	130	112

The CV curves displayed in Fig. 2 (curves a) recorded using both Au and Pt electrodes show well-defined oxidation (E_ox_) and reduction peaks (E_red_) along with good peak-to-peak separation values. The corresponding oxidation peak current and reduction peak current values are designated as I_p_^ox^ and I_p_^red^. The respective E_ox_ and E_red_ values for Au electrode in Fe^2+/3+^ redox system in absence of bio-mimics are determined to be 141 mV and 5 mV along with E_1/2_ value of 136 mV. Similarly the corresponding I_p_^ox^ and I_p_^red^ values are found to be 5.5 × 10^−5^A and − 5.2 × 10^−5^A. In case of Pt electrode, E_ox_ and E_red_ values are calculated to be 119 mV and 12 mV along with E_1/2_ value of 107 mV respectively and the corresponding I_p_^ox^ and I_p_^red^ values are found to be 5.8 × 10^−5^A and − 5.7 × 10^−5^A. The E_1/2_ value of redox couple on Au electrode is found to be relatively higher when compared to Pt electrode. Similarly the respective peak current values are noted to be lower for Au electrode when compared to Pt electrode for the redox system.

Similarly in case of Au electrode, E_ox_ and E_red_ values measured for Fe^2+/3+^ redox probe along with sweat solution are measured to be 135 mV and 02 mV respectively possessing E_1/2_ value of 133 mV. The corresponding I_p_^ox^ and I_p_^red^ values are displayed in Table 1. On the other hand, in case of Pt electrode, E_1/2_ value of 117 mV is determined. The corresponding E_ox_ and E_red_ values for Fe^2+/3+^ redox system along with I_p_^ox^ and I_p_^red^ values are shown in Table 1. From these values, it is noted that there is a marginal shift is observed in E_ox_ and E_red_ peak values associated with the redox probe for both Au and Pt electrodes in artificially simulated sweat composition. The reason for this potential shift is attributed to pH of the sweat solution which is about 6.6, related to the decrease in number of hydroxyl groups when compared to pH of the redox probe (7.3) in absence of sweat composition, thus leading to a very minimal shift in the potential values for Au electrode.

Cyclic voltammograms displayed in Fig. 2C and D correspond to the response obtained for Fe^2+/3+^ redox system in absence (curves a) and in presence (curves b) of artificially simulated saliva compositions using Au and Pt electrodes respectively. Formation of well resolved redox peaks suggests the occurrence of electron transfer reaction on these electrode surfaces and it is controlled by diffusion process. Various parameters such E_ox_ and E_red_ values of the redox probe in saliva solution and the corresponding I_p_^ox^ and I_p_^red^ values along with E_1/2_ values are shown in Table 1. These parameters are determined from CV curves recorded using both Au and Pt electrodes. Interestingly in case of saliva solution both Au and Pt electrodes display a positive shift in peak potential values. From these CV curves it can be understood that both Au and Pt electrodes display a good redox behaviour for Fe^2+/3+^ system in presence of artificially simulated sweat and saliva compositions. Though the redox current values are more or less same, the potential is shifted to positive potential values and it is essentially attributed to change in pH value. Further real sample analysis experiments are performed by collecting sweat sample from healthy individuals and the electron transfer characteristics are analyzed using CV studies. It can be seen from Table 1 that the values obtained for such real sweat sample matches very well with the artificially simulated samples. This observation validates our experimental results and the characteristic parameters determined from CV studies represent the electron transfer reactions occurring in human body fluids. Nevertheless these results clearly show that the electron transfer reaction of a redox probe is retained in presence of sweat and saliva compositions and could be monitored using CV.

**Figure 3:**
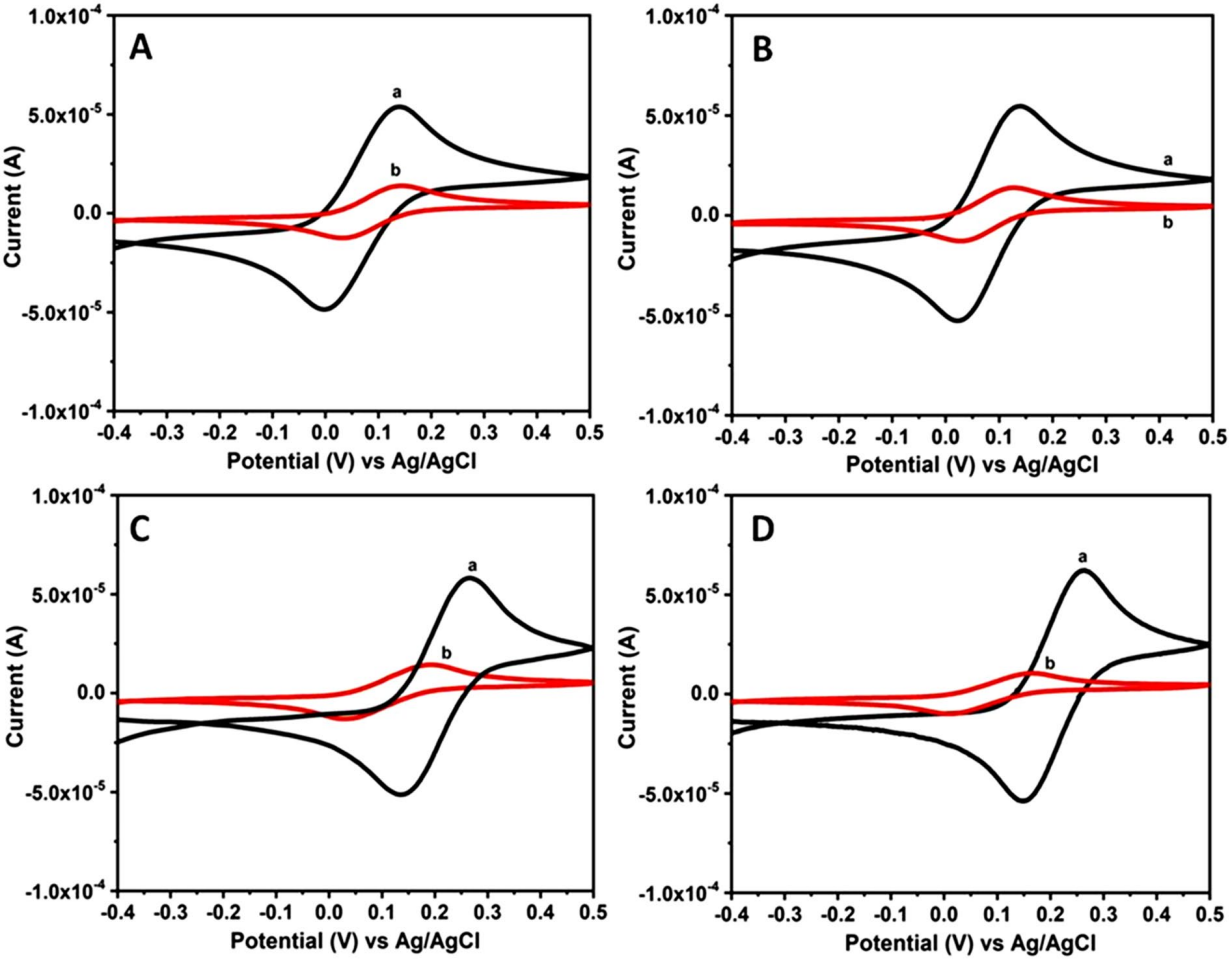
CV curves of Au and Pt electrodes recorded using sweat and saliva bio-mimic solutions at a fixed scan rate of 50 mV/s. (**A**) Au and (**B**) Pt electrodes in artificially simulated sweat solution + Fe^2+/3+^ redox couple + Triton X-100. Similarly (**C**) Au and (**D**) Pt electrodes in artificial saliva solution + Fe^2+/3+^ redox couple + Triton X-100. These CVs are recorded for 1 mM [Fe(CN)_6_]^3−/4−^ redox couple containing 0.1 M NaCl in absence (a) and in presence (b) of Triton X-100 composition respectively.

Similarly Fig. 3A–D shows the CV curves recorded using Au and Pt electrodes in water, Triton X-100 composition consisting of 1 mM [Fe(CN)_6_]^3−/4−^ in absence (Fig. a) and in presence of artificially simulated either sweat or saliva solutions (Fig. b). These studies were carried out within the potential range of − 0.4 to + 0.5 V (vs. Ag/AgCl) at a fixed scan rate of 50 mV/s. These voltammograms display a clear peak formation associated with Fe^2+/3+^ redox couple system suggesting a reversible and also diffusion controlled process of the redox probe. In case of Au electrode studied in Fe^2+/3+^ redox probe with sweat solution containing Triton X-100 composition, the E_ox_ and E_red_ values are determined to be 144 mV and 28 mV along with E_1/2_ value of 116 mV respectively. The corresponding I_p_^ox^ and I_p_^red^ values are shown in Table 1. Similarly, E_ox_, E_red_ I_p_^ox^ and I_p_^red^ along with E_1/2_ values obtained for Pt electrode in Fe^2+/3+^ redox system are displayed in Table 1. It can be noticed from these CVs, a drastic reduction in the current values and much smaller difference in E_1/2_ values for the redox probe in case of lyotropic liquid crystalline phase when compared to sweat solution. On the other hand, in case of saliva system comprising of Triton X-100, E_p_^ox^ peak shifted from 265 to 191 mV and E_p_^red^ peak shifted from 134 to 33 mV for Au electrode, as observed from CVs shown in Fig. 3C. The corresponding I_p_^ox^ and I_p_^red^ values are presented in Table 1. Similarly E_p_^ox^ peak is shifted from 261 to 164 mV and E_p_^red^ peak shifted from 147 to 10 mV along with the E_1/2_ value of 154 mV for Pt electrode in the presence of Triton X-100 and saliva solution, as evident from Fig. 3D. The corresponding I_p_^ox^ and I_p_^red^ values are given in Table 1 for comparison. It can be noticed from these CV data that bio-mimicking systems comprising of either sweat or saliva showed a significant decrease in the redox peak current values in presence of a hexagonal liquid crystalline phase. The reason for this behaviour is mainly attributed to slower diffusion of the redox probe arising out of hexagonally ordered lyotropic structure formed using Triton X-100 and water compositions (Fig. 1B,C) which results in blocking of the electron transfer reaction and ultimately the reduction in current values.

**Figure 4:**
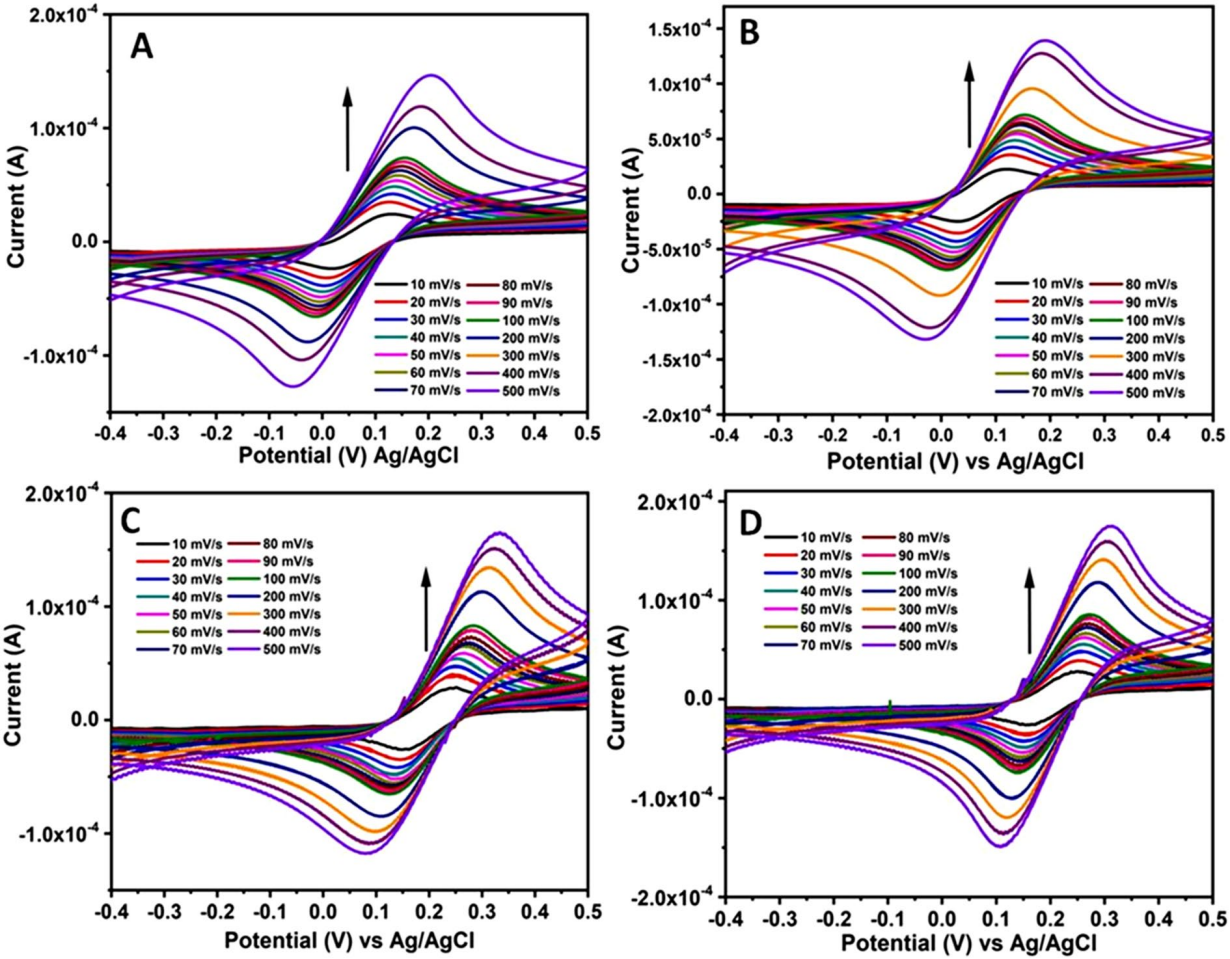
CV curves recorded for Au and Pt electrodes using sweat and saliva bio-mimic solutions over a wide range of scan rate from 10 to 500 mV/s. (**A**) Au and (**B**) Pt electrodes studied in artificially simulated sweat solution + Fe^2+/3+^ redox couple. Similarly (**C**) Au and (**D**) Pt electrodes investigated in artificial saliva solution + Fe^2+/3+^ redox couple. These CVs are recorded for 1 mM [Fe(CN)_6_]^3−/4−^ redox couple containing 0.1 M NaCl as the supporting electrolyte. In these figures arrows indicate the direction of increasing scan rate.

In addition the effect of sweep rate on the electron transfer characteristics is also investigated by recording the corresponding CV responses over a wide range of scan rate from 10 to 500 mV/s. These CV curves are displayed in Fig. 4. Initially the effect of scan rate was recorded for [Fe(CN)_6_]^3−/4−^ redox couple using Au and Pt electrodes in presence of either sweat or saliva compositions (Fig. 4A–D). In this figure, the arrows indicate the direction of increasing scan rate. It can be observed from these CVs that the peak current values for both the oxidation and reduction processes increase with respect to increasing scan rate. Moreover, the shape of these CV curves along with the peak potential separation values suggest a perfect diffusion controlled process for the redox couple in these sweat and saliva bio-mimics. Similarly the effect on scan rate is also investigated using the hexagonal lyotropic liquid crystalline phase by employing sweat and saliva compositions and the corresponding CV curves were shown in supporting information (ESI; Fig. S2). These CV curves also exhibit increasing peak current values with respect to increasing scan rate. Further the separation between the peak potential values and the shape of CV curves indicate the perfect diffusion controlled process for the reversible system.

**Figure 5:**
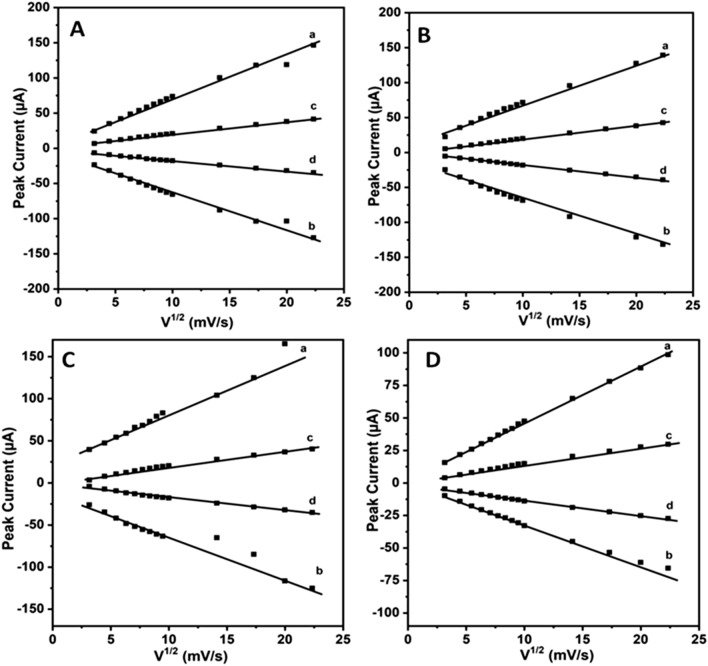
Plots of peak current vs. square root of scan rate obtained using Au (**A**,**C**) and Pt (**B**,**D**) electrodes for various bio-mimic systems studied in this work namely (**A**) Sweat, (**B**) Saliva, (**C**) Sweat + Triton X-100 and (**D**) Saliva + Triton X-100 respectively. These data points were collected from Figs. 4 and S2. In these plots (a) & (c) represent I_p_^ox^ versus ν^1/2^ and (b) & (d) designate I_p_^red^ versus ν^1/2^ respectively.

In order to verify further the reversibility and diffusion controlled nature of the redox probe, a plot of peak current versus the square root of scan rate is drawn. The data points were collected from CV curves displayed in Figs. 4 and S2. These CV curves were recorded over a wide range of scan rate covering at least two orders of magnitude. Figure 5A–D shows the plots of peak currents (obtained for both oxidation and reduction processes) vs. square root of scan rate for all the systems investigated in this work viz., sweat, saliva, sweat + liquid crystalline phase and saliva + liquid crystalline phase respectively consisting of the redox couple. These data points were collected using Au and Pt electrodes. From these plots, useful information on kinetics and the mechanism behind the electron transfer reaction could be obtained. It can be observed from these plots a perfect linear relation between the peak current and square root of scan rate value, suggesting a complete diffusion controlled process for the reversible electron transfer reaction. This follows a typical Randles–Sevcik behaviour and from the slope values of these plots, we can determine the diffusion coefficient values using Eq. (1) shown below.

1 $${I}_{p}=(2.69 X {10}^{5}){ C}_{O}{n}^{3/2}{\gamma }^{1/2}{D}^{1/2}A$$

where I_p_ is peak current in ampere, C_O_ is concentration of the redox probe in mol/L, n is the number of electrons, D is diffusion coefficient in cm^2^/s, $\gamma$ is scan rate in mV/s and A is the electrode surface area in cm^2^ respectively.

From these plots, it can be noted that the peak current increases linearly with square root of scan rate for all the systems indicating a perfect diffusion-controlled process for the reversible electron transfer reaction. By analyzing the slope values of these plots and by using Eq. (1), diffusion coefficient values were calculated for all the systems studied in this work and they are shown in Table 2.

**Table 2:** Diffusion coefficient values of the redox probe obtained from the slope values of the plots shown in Fig. 5 for all the systems investigated in this work using Au and Pt electrodes.

Electrolyte	Au electrode (× 10^−6^ cm^2^/s)		Pt electrode (× 10^−6^ cm^2^/s)	
	Oxidation	Reduction	Oxidation	Reduction
Fe^2+/3+^	5.78	2.75	5.95	5.56
Sweat + Fe^2+/3+^	4.70	5.59	4.16	3.68
Sweat + Fe^2+/3+^ + Triton X-100	0.38	0.25	0.41	0.33
Saliva + Fe^2+/3+^	3.61	0.26	1.96	0.96
Saliva + Fe^2+/3+^ + Triton X-100	0.36	0.26	0.19	0.16
Real sweat sample analysis	4.63	5.52	4.12	3.59

It can be observed from Table 2 that the diffusion coefficient values of [Fe(CN)_6_]^3−/4−^ redox couple agrees very well with the literature reports^30–33^. These values remain more or less similar in the case of sweat and saliva bio-mimics. In addition, the diffusion coefficient value obtained for real sample analysis carried out using sweat sample collected from healthy individual is also shown and particularly this value matches very well with the artificially simulated sweat solution and thus validating our experimental results. On the other hand, when liquid crystalline phase is used for the study along with either sweat or saliva compositions, the diffusion coefficient values decreased by an order of magnitude. This may be due to the slower diffusion of redox probe molecule within the ordered liquid crystalline system^36^. Nevertheless these results clearly show that both sweat and saliva bio-mimics could well be used for the study of electron transfer reactions. Further these systems also offer an opportunity to modulate such redox processes for effective understanding of sensing and catalytic applications.

### Electrochemical impedance spectroscopy

**Figure 6:**
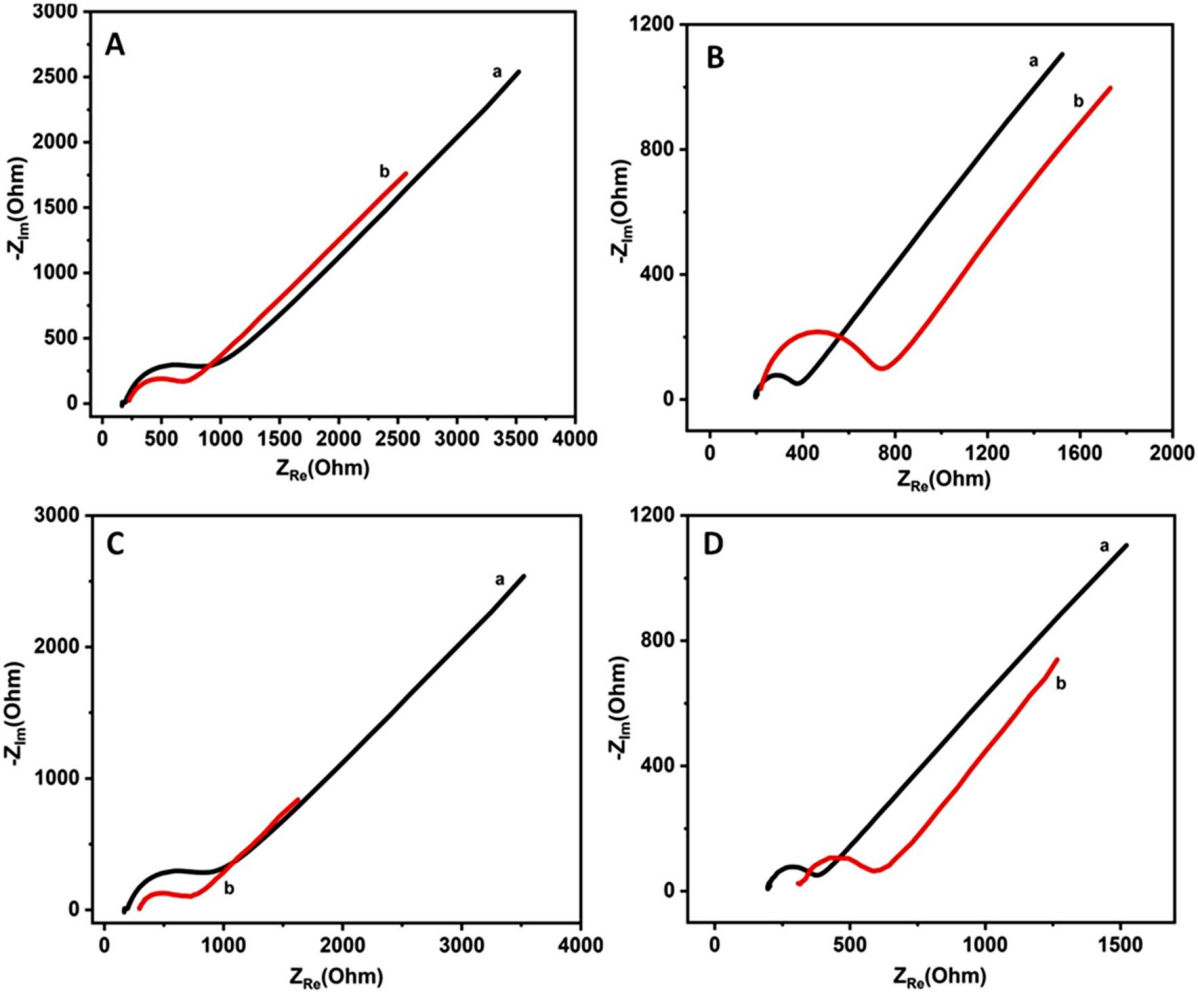
Nyquist plots of Au and Pt electrodes recorded using sweat and saliva bio-mimic solutions at half-wave potentials (E_1/2_). (**A**) Au and (**B**) Pt electrodes’ impedance responses obtained in artificially simulated sweat solution + Fe^2+/3+^ redox couple. Similarly (**C**) Au and (**D**) Pt electrodes’ impedance data recorded in artificial saliva solution + Fe^2+/3+^ redox couple. These curves are recorded using 1 mM [Fe(CN)_6_]^3−/4−^ redox couple containing 0.1 M NaCl in absence (a) and in presence (b) of either sweat or saliva compositions respectively.

Electrochemical impedance spectroscopic measurements were performed to investigate the electron transfer reaction of [Fe(CN)_6_]^3−/4−^ redox couple and to further determine the kinetic parameters such as charge transfer resistance and electron transfer rate constants using sweat and saliva bio-mimics. These experiments were carried out at E_1/2_ value of the redox couple using 1 mM potassium ferro/ferri cyanide solution consisting of 0.1 M NaCl as a supporting electrolyte. A small sinusoidal voltage of 5 mV was applied over a wide frequency ranging from 100 kHz to 100 mHz. The corresponding impedance results were represented as Nyquist plots shown in Fig. 6A–D.

Figure 6A and B show the Nyquist plots obtained for the redox couple dissolved in artificial sweat composition using Au and Pt electrodes respectively. Similarly, Fig. 6C and D display the impedance plots recorded in artificial saliva composition consisting of Fe^2+/3+^ redox couple using Au and Pt electrodes. In these plots curve (a) denotes the impedance response of the redox couple in absence of either sweat or saliva compositions and (b) represents the similar impedance plots obtained in presence of such artificially simulated sweat or saliva bio-mimics. These plots exhibit a small semicircle at higher frequency region and a straight line at low frequency region, suggesting a perfect diffusion controlled process for the electron transfer reaction. In general, the formation of a semicircle signifies a charge transfer controlled process; and in contrast the straight inclined line designates a diffusion controlled process. The parameter, charge transfer resistance (R_ct_) is determined by fitting the higher frequency region to a semicircle and from the diameter by calculating the intercept on the x-axis of Nyquist plot. R_ct_ represents the resistance offered by the electrode towards the electron transfer reaction. On the other hand, resistance to the mass transfer (diffusion) is denoted by Warburg impedance (W), as a straight line. In case of Au electrode, R_ct_ is determined to be 722.2 Ω for Fe^2+/3+^ redox couple in absence of bio-mimic solution and this value is significantly reduced to 377.8 Ω for sweat and 442.4 Ω for saliva compositions. Similarly, Pt electrodes exhibit R_ct_ value of 170.6 Ω for the redox couple in absence of the artificial bio-mimics and this value is drastically changed to 488.8 Ω for sweat and 275.7 Ω for saliva compositions respectively.

**Figure 7:**
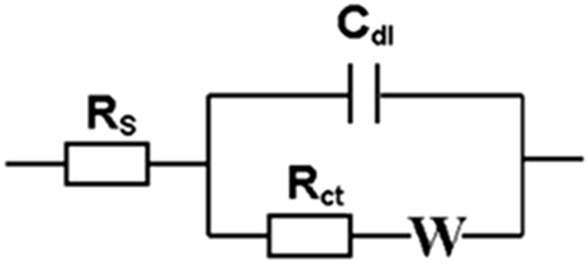
Randles equivalent circuit model used for fitting the measured impedance data and subsequently to determine the kinetic parameters.

Similarly, the charge transfer resistance values could also be determined by fitting the measured impedance data to an equivalent circuit. The most common Randles equivalent circuit (modified) shown in Fig. 7 is used for this purpose. It can be seen from this figure that, this particular equivalent circuit has several elements namely the solution resistance (R_s_) connected in series to a parallel combination of double layer capacitance (C_dl_) and charge transfer resistance (R_ct_) along with Warburg impedance (W) connected in series to it. In some cases especially where a depressed semicircle is observed, instead of C_dl_, a constant phase element (Q) is used in the equivalent circuit model and W accounts for the resistance offered towards the mass transfer process, the diffusion of redox probe. This equivalent circuit is used for the determination of R_ct_ associated with various systems studied in this work and those values are given in Table 3.

**Table 3:** Charge transfer resistance (R_ct_) and electron transfer rate constant (K_et_) values obtained using Au and Pt electrodes studied in artificially simulated sweat and saliva bio-mimic solutions consisting of hexagonal lyotropic liquid crystalline phase (Triton X-100) with Fe^2+/3+^ redox couple.

Electrolyte	Charge transfer resistance (R_ct_) (Ω)		Rate constant (K_et_) (cm/s)	
	Au	Pt	Au	Pt
Fe^2+/3+^	722.1	170.6	0.52	2.20
Sweat + Fe^2+/3+^	377.8	488.8	0.99	0.76
Sweat + Fe^2+/3+^ + Triton X-100	1119	912.8	0.33	0.04
Saliva + Fe^2+/3+^	442.4	275.7	0.85	1.36
Saliva + Fe^2+/3+^ + Triton X-100	12,730	1370	0.03	0.27
Real sweat sample analysis	372.4	481.5	0.91	0.69

The electron transfer rate constant (K_et_) values are calculated from the R_ct_ values determined by equivalent circuit fitting using Eq. (2), shown below.

2 $${K}_{et}= \frac{RT}{{n}^{2}{F}^{2}A{C}_{0}{R}_{ct}}$$

where R is gas constant (8.314 J/K mol), T is temperature in K, n is the number of electrons, F is Faraday’s constant (96,485 C/mol), A is the electrode surface area in cm^2^, C is the concentration of redox probe in mol/L and R_ct_ is the charge transfer resistance in Ω respectively. Using Eq. (2) and R_ct_ values determined from impedance measurements, the electron transfer rate constant values were calculated for various systems investigated in this work and they are shown in Table 3. For instance, in case of Au electrode, K_et_ value of 0.520 cm/s is obtained in absence of bio-mimics for the redox couple and this value has slightly been enhanced to 0.990 cm/s and 0.85 cm/s in presence of sweat and saliva compositions respectively. These values correlate very well with the observed changes in R_ct_ values.

Furthermore, similar impedance measurements were also carried out using artificially simulated sweat and saliva bio-mimics in presence of hexagonal lyotropic liquid crystalline phase consisting of Triton X-100 along with Fe^2+/3+^ redox couple by employing Au and Pt electrodes. The corresponding Nyquist plots are shown in supporting information (ESI; Fig. S3). These plots clearly show the formation of a small semicircle at high frequency region and a straight line at low frequency regime for both Au & Pt electrodes in absence of bio-mimics solution indicating a perfect diffusion controlled process for the reversible electron transfer reaction. On the other hand, in presence of hexagonal lyotropic liquid crystalline phase with artificially simulated either sweat or saliva compositions, the impedance response exhibits a higher R_ct_ values suggesting a charge transfer controlled process for the redox couple. Concomitantly the electron transfer rate constant values are also significantly decreased. These values are provided in Table 3 for comparison. One of the interesting features noted in these impedance plots is the shift of R_s_ values and it is mainly attributed to the solution compositions arising out of sweat and saliva bio-mimic solutions along with the respective change in pH values. It is evident from these studies that the hexagonal liquid crystalline phase consisting of Triton X-100 exhibits a blocking behaviour by providing a barrier layer and inhibits the electron transfer reaction, which is manifested in increased R_ct_ and decreased K_et_ values. These results agree very well with CV data discussed earlier.

Overall, our studies clearly demonstrate the possibility of investigating and at the same time modulating the electron transfer reaction of a redox probe in artificially simulated sweat and saliva bio-mimic solutions. This study becomes more important due to the recent developments of biosensors for non-invasive analysis using human body fluids like sweat, saliva and tear along with a redox probe for specific detection of target analytes. In this context, our results offer an opportunity to impart direct electron transfer studies of a well-known redox probe and utilizing them for the analysis and for the subsequent determination of kinetic parameters. Nevertheless, this work could also contribute to the fundamental understanding of electron transfer reaction investigated using the unconventional electrolytes.

## Experimental section

### Chemicals

All the chemicals used in this work were of analytical reagent grade. Millipore water having a resistivity of 18.2 MΩ cm obtained from Merck millipore water unit was used for the preparation of all the aqueous solutions employed for electrochemical studies. Sodium chloride (Merck), lactic acid (Merck) and urea (Fischer Scientific) were used for the preparation of sweat bio-mimic compositions. Similarly, methyl-p-hydroxybenzoate (Avra), sodiumcarboxymethyl cellulose (Sigma Aldrich), potassium chloride (Alfa Aesar), magnesium chloride (Merck), calcium chloride (Fischer Scientific), dipotassium hydrogen phosphate (Merck) and potassium dihydrogen phosphate (Hi-Media) were utilized for the preparation of saliva bio-mimic solutions. Electrochemical studies were carried out using potassium ferricyanide, K_3_[Fe(CN)_6_] (Fischer Scientific) and potassium ferrocyanide, K_4_[Fe(CN)_6_] (Hi-Media) as the redox probe. Finally Triton X-100 (Sisco Research Laboratories) was used to prepare the hexagonal lyotropic liquid crystalline medium. All these chemicals were used as received without any further purification.

### Preparation of artificial sweat and saliva bio-mimics

Typical compositions used for the preparation of sweat and saliva bio-mimic solutions were shown in Table 4. In case of sweat, sodium chloride (5 g), urea (1 g) and lactic acid (1 g) were dissolved in 1 L of millipore water. Then pH of this artificial sweat solution was adjusted to 6.6 using ammonium hydroxide as per the standard procedure established by British-Adopted European Standard^39^. Similarly, the artificial saliva solution was prepared according to Macknight Hane and Whitford formulation^40^. Initially 2 g of methyl-p-hydroxybenzoate was dissolved in 800 mL of millipore water and 20 mL of this solution was used as a stock solution while the remaining solution was stored in a refrigerator. Then 10 g of sodium carboxymethyl cellulose was dissolved in 200 mL of boiling water by continuous stirring until total sodium carboxymethyl cellulose was dissolved. Afterwards 800 mL of cold methyl-p-hydroxybenzoate solution was poured into sodium carboxymethyl cellulose solution and then mixed together resulting in the formation of a gel. About 0.625 g of potassium chloride was dissolved in 2 mL of methyl-p-hydroxybenzoate solution and then poured into the previously prepared gel solution. The resultant solution was again mixed with 0.059 g of magnesium chloride and 0.166 g of calcium chloride in 2 mL of methyl-*p*-hydroxybenzoate solution. Afterwards about 0.804 g of dipotassium hydrogen phosphate and 0.326 g of potassium dihydrogen phosphate were also added to the above mixture. Finally the artificial saliva bio-mimic composition was prepared by thoroughly mixing these solutions. Later on, pH of this saliva solution was adjusted to 6.2 using aqueous KOH solution.

**Table 4:** Chemical compositions of artificially simulated sweat and saliva bio-mimic solutions (per liter).

Sweat compositions	Saliva compositions
Sodium chloride—5 g Lactic acid—1 g Urea—1 g	Methyl-p-hydroxybenzoate—2 g Sodium carboxymethyl cellulose—10 g KCl—0.625 g MgCl_2_.6H_2_O—0.059 g CaCl_2_.2H_2_O—0.166 g K_2_HPO_4_—0.804 g KH_2_PO_4_—0.326 g

Interestingly these bio-mimics were used as the electrolyte for investigating electron transfer reaction of a redox probe namely, [Fe(CN)_6_]^3−/4−^ couple. In addition, similar electron transfer characteristics were also studied using lyotropic liquid crystalline phase formed with the help of 52% (wt.) water and 48% (wt.) Triton X-100 compositions. Here, the aqueous phase was replaced with artificial sweat and saliva bio-mimic solutions consisting of the redox probe. It is worth mentioning here that even after replacement, the resultant mixtures exhibit and retain the lyotropic liquid crystalline phase, as confirmed from POM studies (Fig. 1).

### Electron transfer studies using unconventional electrolytes

Electrochemical techniques namely cyclic voltammetry (CV) and electrochemical impedance spectroscopy (EIS) were used for the investigation. Typically electron transfer characteristics of potassium ferro/ferri cyanide redox couple were studied using artificially simulated sweat and saliva compositions as the unconventional electrolytes. These studies were carried out using either gold (Au) or platinum (Pt) electrodes as the working electrode, Ag/AgCl as the reference and Pt wire as the counter electrodes respectively. The electrochemical techniques are recognized as highly desirable methods for the detection of biomolecules because of the use of small electrode size, real-time and direct analysis with high sensitivity and selectivity towards the target analytes present as a potential biomarker in these bio-fluids^41^. Among the different electrodes, Au and Pt are the most popular and widely used electrodes to investigate the electron transfer reaction. Moreover these two electrodes offer a wide range of potential window for the study and exhibit better stability when compared to other electrodes^41^. Based on this observation we have selected 3 mm diameter size of disc shaped Au and Pt electrodes for our studies. Prior to the analysis, these electrodes were polished to a mirror-like surface with progressively decreasing alumina slurries viz., 1 μm, 0.3 μm and 0.05 μm. The polished electrodes were thoroughly rinsed with millipore water between each polishing step. Then it was washed successively with millipore water in an ultrasonic bath and dried in air at room temperature.

In a typical experiment, CV studies were performed within the potential range from − 0.4 V to 0.5 V at a fixed scan rate of 50 mV/s. The electrolyte consisting of either artificially simulated sweat or saliva composition along with 1 mM potassium ferro/ferri cyanide as a redox couple was used for the study. Moreover, the effect of scan rate on electron transfer characteristics was also investigated over a wide range of scan rate from 10 mV/s to 500 mV/s. Further impedance measurements were carried out using these electrolytes at a fixed half-wave potential (E_1/2_) over a wide range of frequency from 100 kHz to 100 mHz by applying 5 mV sinusoidal peak to peak perturbation. For comparison many different control experiments in presence of lyotropic liquid crystalline phase, simple aqueous medium, in absence of sweat or saliva compositions and in absence of hexagonal liquid crystalline phase were also performed. Several parameters including diffusion coefficient (D), charge transfer resistance (R_ct_) and electron transfer rate constant (K_et_) values are determined and compared. These electrochemical experiments were carried out using Bio-Logic (Model: SP-240) equipment procured from France. EC lab software provided by them was used for the analysis of data.

## Conclusions

In this work electron transfer reaction of a redox probe namely, potassium ferro/ferri cyanide dissolved in unconventional electrolytes made up of sweat and saliva bio-mimics constituents is investigated using electrochemical techniques like cyclic voltammetry and electrochemical impedance spectroscopy. Further the effect of hexagonal lyotropic liquid crystalline phase formed using these bio-mimics on the electron transfer reaction is also studied. It has been concluded from CV and EIS results that the electron transfer reaction associated with a redox couple, [Fe(CN)_6_]^3−/4−^ is completely under diffusion controlled process and interestingly this is not altered much in the case of sweat and saliva bio-mimics being used as the electrolytes for the studies. On the other hand, the electron transfer reaction is completely under charge transfer controlled when the liquid crystalline phase is employed as the electrolytic medium. This effect is even more pronounced in case of sweat and saliva bio-mimics being used along with the hexagonal lyotropic liquid crystal. Many different kinetic parameters including diffusion coefficient values, charge transfer resistance and electron transfer rate constant values are determined and compared for the analysis. These results clearly reveal that sweat and saliva could be used as the unconventional electrolytes for understanding the electron transfer reactions and subsequently could be utilized for modulation of such electron transfer processes for applications in the fields of bio-sensors and electrocatalysis.

## Supplementary Information

Supplementary Information
