# Supplementary material for: Electron transfer studies of a conventional redox probe in human sweat and saliva bio-mimicking conditions
Source: Sci Rep. 2021 Apr 7;11:7663. doi: 10.1038/s41598-021-86866-z (PMC8027883; doi:10.1038/s41598-021-86866-z)
Supplement: Supplementary file 1 — Supplementary Information [file 41598_2021_86866_MOESM1_ESM.pdf]

Electronic Supporting Information (ESI) File:

Electron Transfer Studies of a Conventional Redox Probe in Human Sweat and Saliva Bio-mimicking Conditions

P. Krishnaveni[a] and V. Ganesh[a,b,*]

[a] *Electrodics and Electrocatalysis (EEC) Division, CSIR – Central Electrochemical Research Institute (CSIR – CECRI), Karaikudi – 630003, Tamil Nadu, India.*

[b]*Academy of Scientific and Innovative Research (AcSIR), Ghaziabad – 201002, India.*

* Corresponding Author's E-mail: vganesh@cecri.res.in (or) ganelectro@gmail.com

Tel: +91-4565-241242; Fax: +91-4565-227779.

Figures:

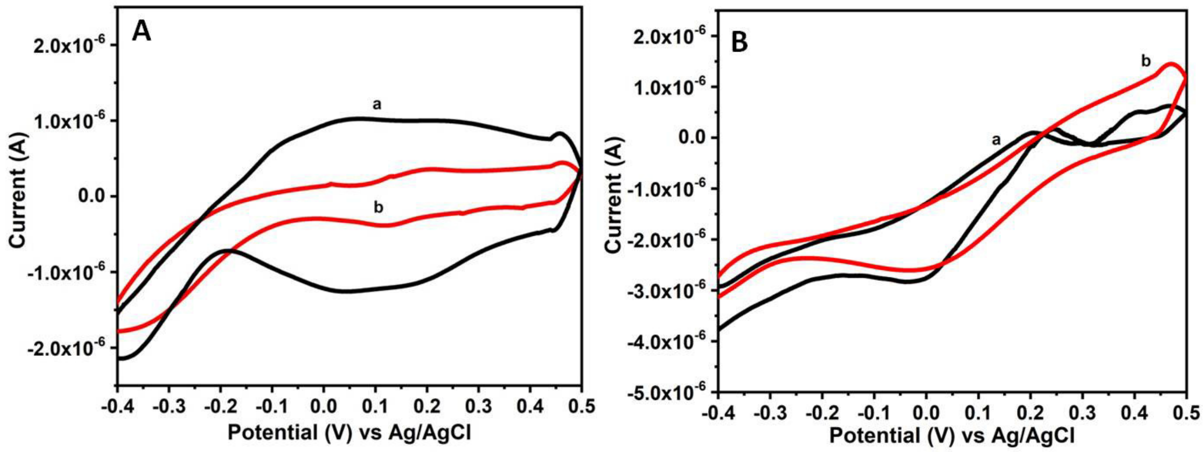

Fig. S1: CV curves of (A) Au and (B) Pt electrodes recorded using artificially simulated sweat (a) and saliva (b) compositions in absence of redox probe at a fixed scan rate of 50 mV/s.

The effect of sweep rate on the electron transfer characteristics is investigated by recording the corresponding CV responses over a range of scan rate from 10 mV/s to 500 mV/s using Au and Pt electrodes in sweat and saliva solution along with 1 mM $[Fe(CN)_6]^{3-/4-}$ / Triton X-100. The recorded CVs are shown in Figure S2 (A-D).

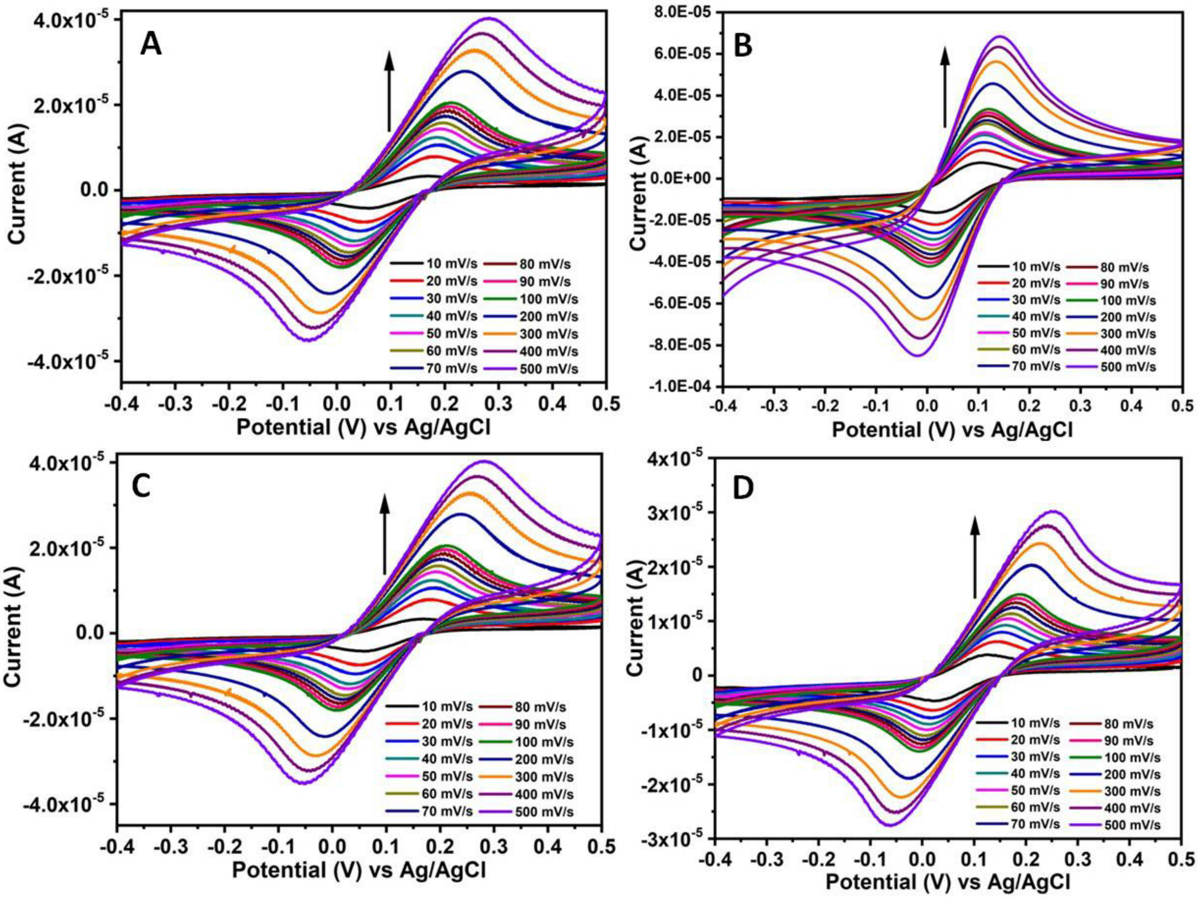

Fig. S2: CV curves of Au and Pt electrodes recorded using sweat and saliva bio-mimic solutions over a wide range of scan rate from 10 mV/s to 500 mV/s. (A) Au and (B) Pt electrodes studied in artificially simulated sweat solution + $Fe^{2+/3+}$ redox couple + Triton X-100. Similarly (C) Au and (D) Pt electrodes investigated in artificial saliva solution + $Fe^{2+/3+}$ redox couple + Triton X-100. These CVs are recorded for 1 mM $[Fe(CN)_6]^{3-/4-}$ redox couple containing 0.1 M NaCl. In these figures arrows indicate the direction of increasing scan rate.

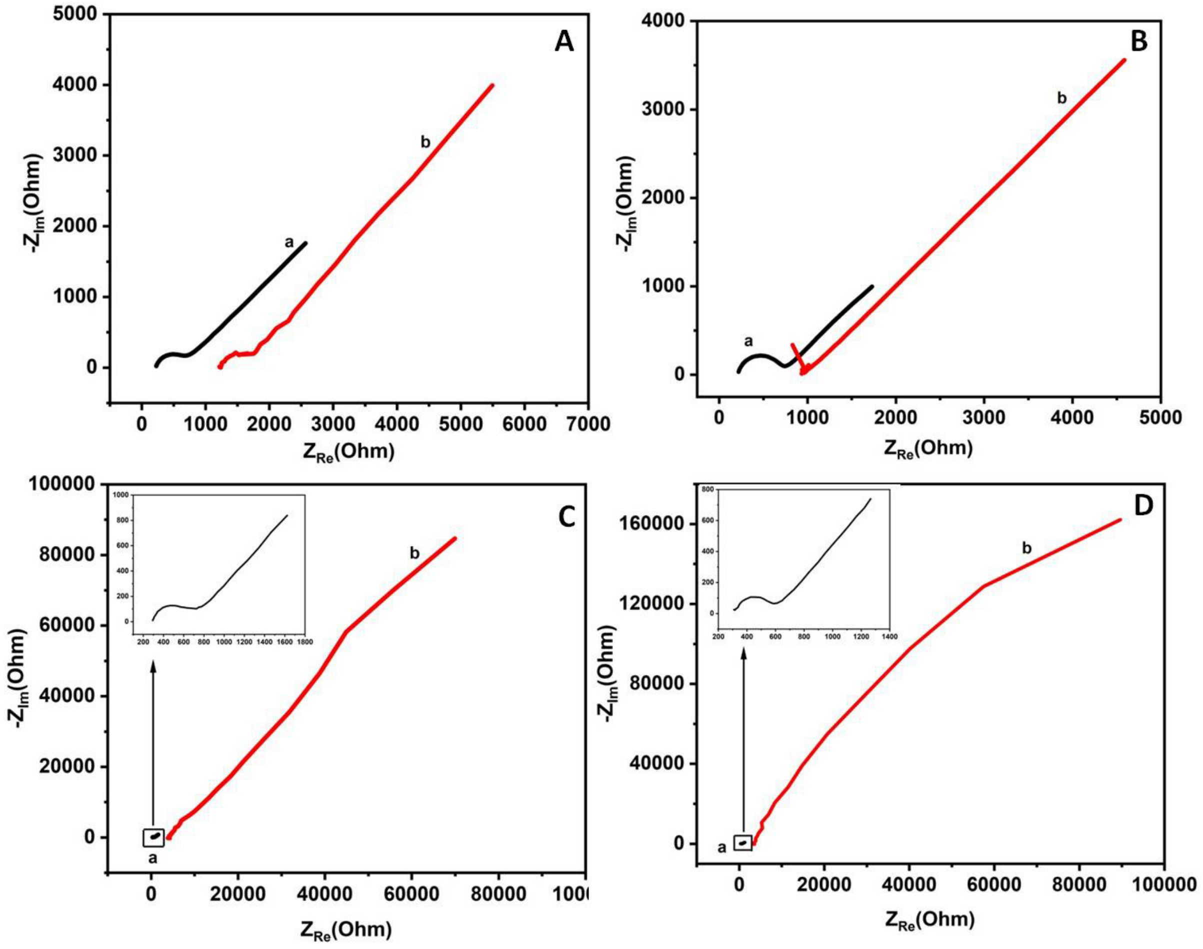

Fig. S3: Impedance plots represented as Nyquist plots of Au and Pt electrodes recorded using sweat and saliva bio-mimic solutions at half-wave potentials ($E_{1/2}$). (A) Au and (B) Pt electrodes' impedance responses obtained in artificially simulated sweat solution + $Fe^{2+/3+}$ redox couple + liquid crystalline phase (Triton X-100). Similarly (C) Au and (D) Pt electrodes' impedance data recorded in artificial saliva solution + $Fe^{2+/3+}$ redox couple + liquid crystalline phase (Triton X-100). These curves are recorded using 1 mM $[Fe(CN)_6]^{3-/4-}$ redox couple containing 0.1 M NaCl in absence (a) and in presence (b) of either sweat or saliva compositions respectively. The insets shown in Figures (C) and (D) correspond to Nyquist plots obtained in absence of either sweat or saliva solutions using Au and Pt electrodes.
